# Lingual Frenotomy in Pediatric Ankyloglossia: A Diode Laser Approach in Two Case Reports

**DOI:** 10.7759/cureus.53701

**Published:** 2024-02-06

**Authors:** Joana M Dias, Elsa Paiva, Ines G Pereira, Henrique C Soares, Cristina Areias

**Affiliations:** 1 Dentistry, Oporto University, Porto, PRT; 2 Pediatric Dentistry, Private Practice, Porto, PRT; 3 Neonatology, Centro Hospitalar e Universitário de São João, Porto, PRT

**Keywords:** breastfeeding medicine, lingual frenotomy, diode laser, malocclusion, tongue-tie, ankyloglossia

## Abstract

Ankyloglossia can be related to a number of complications, such as breastfeeding difficulties or alterations in craniofacial development. Treatment can involve surgery to correct the altered lingual frenulum and can be performed by various techniques. The purpose of this paper is to present two case reports of ankyloglossia in pediatric patients of different ages, the diagnostic criteria, and the treatment decision rationale, which led to a lingual frenotomy performed with a diode laser.

## Introduction

Alterations in the lingual frenulum [1] can have an impact on breast or bottle feeding since birth and can persist during the child's developmental stages, hindering activities such as speech, chewing, swallowing, malocclusion, and gingival recession [2]. This anatomic and functional alteration, termed ankyloglossia (tongue-tie), is classified as a congenital condition that involves a short, tight, or thick lingual frenulum [3].

Currently, there is a lack of agreement about the diagnosis and treatment of ankyloglossia (tongue-tie) [4], which leads to a great difference in the condition’s prevalence between countries (1% to 12.1%) [5]; however, scientific publications on this subject have been recently increasing. In Norway, for example, when comparing figures from 2008 to 2019, there were seven times more diagnoses and 13 times more surgical procedures relating to the lingual frenulum in 2019 than in 2008 [6]; meanwhile, a study in Spain found an ankyloglossia prevalence of 46.3% in neonates [5].

Regarding the anatomy of the lingual frenulum, there are no morphological changes, over time, in its thickness and attachment to the tongue and floor of the mouth [7]. Thus, despite little evidence on when and how to perform surgery on the lingual frenulum, lingual frenotomy/frenectomy for functional limitations should be considered on an individual basis [2]. Laser technology for lingual frenotomies/frenectomies has been used for surgery on the lingual frenulum, with a demonstration of a small working time, reduced pain and discomfort, less postoperative complications, best hemostasis control, no sutures needs and preference for the patient [8].

The role of orofacial muscle (i.e., lips, tongue, and oropharynx) alterations on jaw development has been widely discussed, but there is still no consensus in the scientific literature on whether bad oral habits, mouth breathing, and low tongue posture play a role in the etiology and pathogenesis of malocclusions [9,10]. Despite this, whenever problems associated with malocclusion are identified, they are of considerable importance for the prognosis and must be eliminated to ensure adequate growth.

The aim of this paper is to report the management of ankyloglossia in two patients - one at an early age (one month old) and the other in an older child (eight years old) - with different treatment plans by a multidisciplinary medical team but with some similarities in the operative approach.

## Case presentation

Case report 1: one-month-old infant

A one-month-old Caucasian infant presented to our polyclinic in Matosinhos (Portugal) for an evaluation of the tongue, referred by his pediatrician, who observed breastfeeding difficulties and gastroesophageal reflux in the patient. His mother had a regular pregnancy until 40 weeks and 2 days and a eutocic delivery, with breastfeeding and skin-to-skin contact in the first hour after birth. Since the beginning, the mother reported mamillary trauma and nipple pain during breastfeeding. Being a second-time mother, she felt that something was not right with the latch; nevertheless, she had ample milk supply and, consequently, the weight gain was not a concern.

During the appointment, breastfeeding was observed, and the position and latch were corrected by a Lactation Consultant (IBCLC). Aerophagia was clearly audible, with a snapback, moments of gagging during the milk ejection reflex, and several pauses during the session. The latch remained incorrect after repositioning, with visible muscle compensations by the infant.

**Figure 1:**
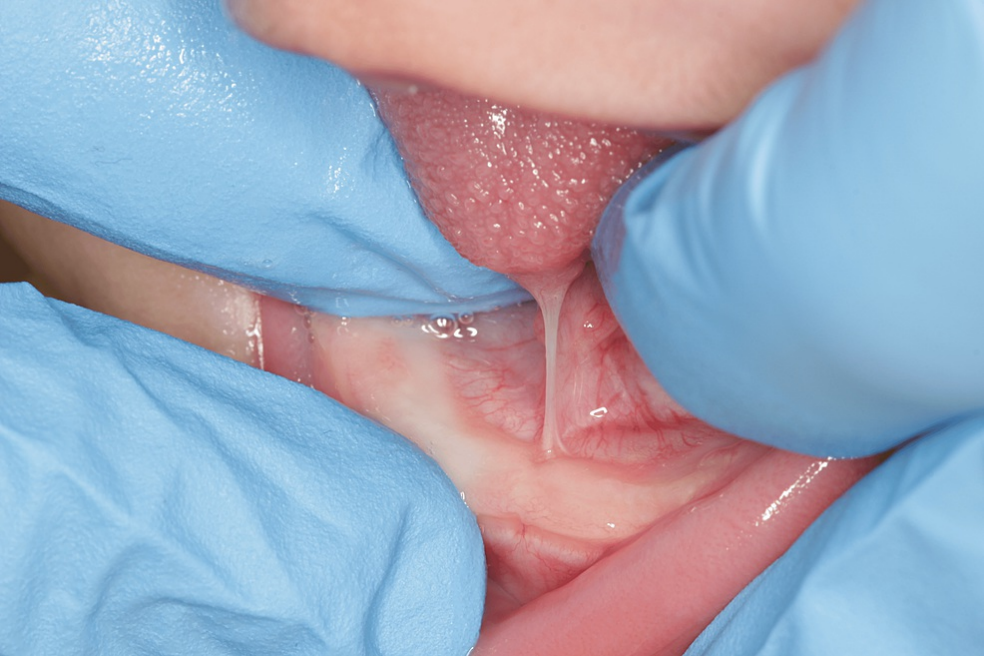
Ankyloglossia (Case 1)

Two protocols for the evaluation of the lingual frenulum were applied: (1) Assessment Tool for Lingual Frenulum Function™ (ATLFF) [11] and (2) Coryllos [12]. This frenulum was classified as ATLFF 7 in function and 5 in appearance, and Coryllos 2, with an indication for surgical treatment (Figure 1).

**Figure 2:**
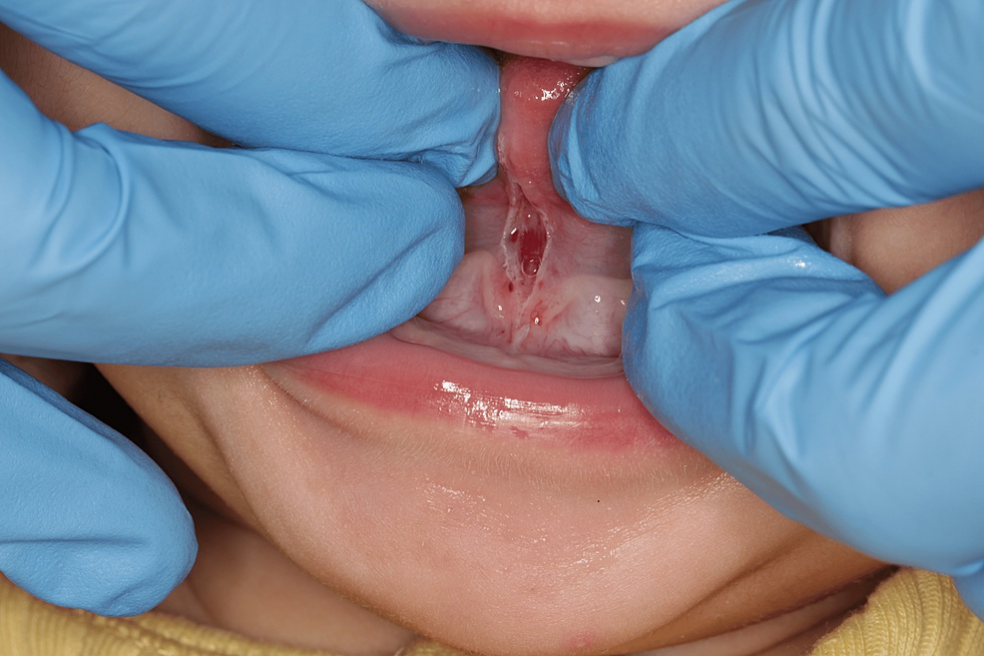
Lingual frenotomy (Case 1)

After discussing all treatment options with the family and obtaining informed consent, lingual frenotomy was scheduled with a simple diode laser technique under local anesthesia [13]: after administering tongue anesthesia with a 2% lidocaine infiltrative solution, the lingual frenulum was dissected from the mucous layer to the fascia. Care was taken to avoid damage to lingual muscles, blood vessels, lingual nerve, and Wharton's duct. Tongue mobility was verified, and a diamond-shaped surgical wound was obtained. Due to the excellent cauterization achieved by the diode laser, no sutures were needed (Figure 2).

**Figure 3:**
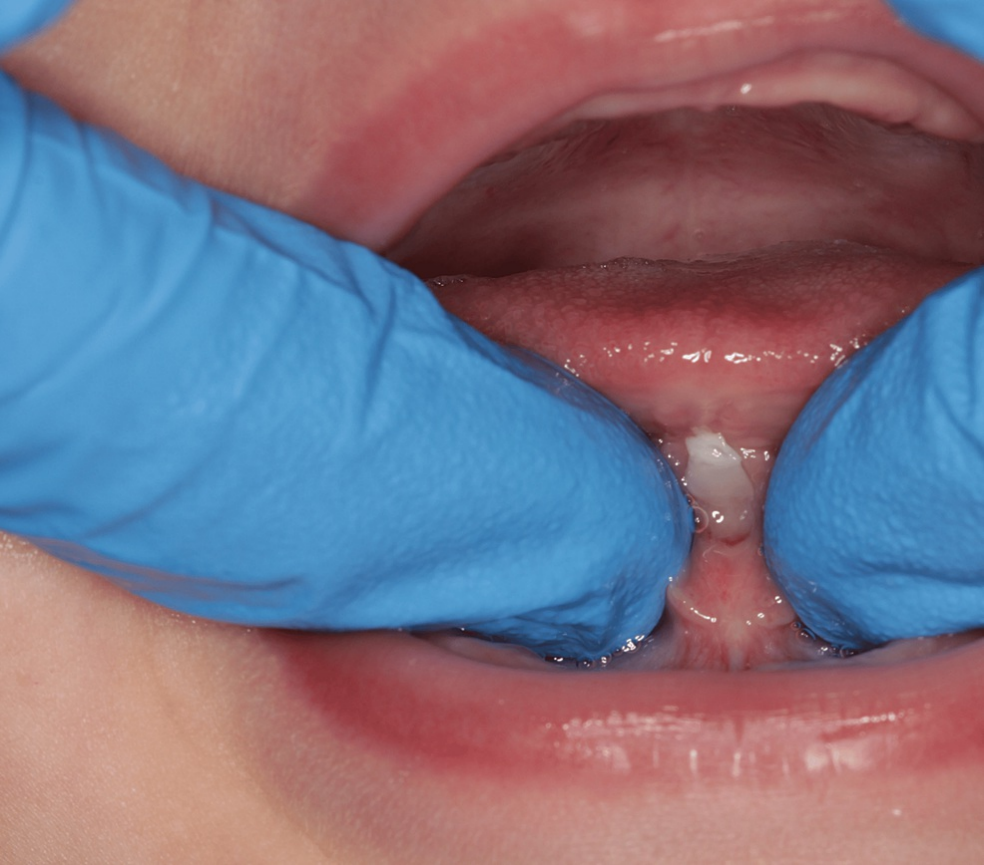
One week post-op (Case 1)

Breastfeeding on cue and analgesic medication for the first two days (paracetamol per os) were recommended. There were no complications, and a follow-up appointment was scheduled eight days after surgery (Figure 3) and the patient was referred to a speech therapist to facilitate better healing and lingual function.

Case report 2: eight-year-old boy

**Figure 4:**
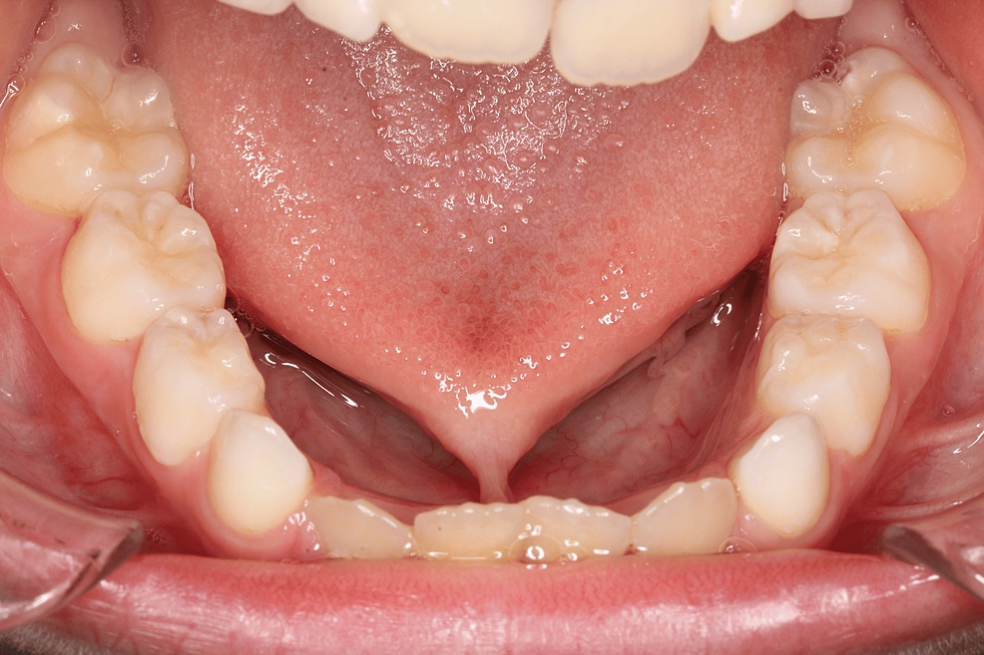
Ankyloglossia (Case 2)

**Figure 5:**
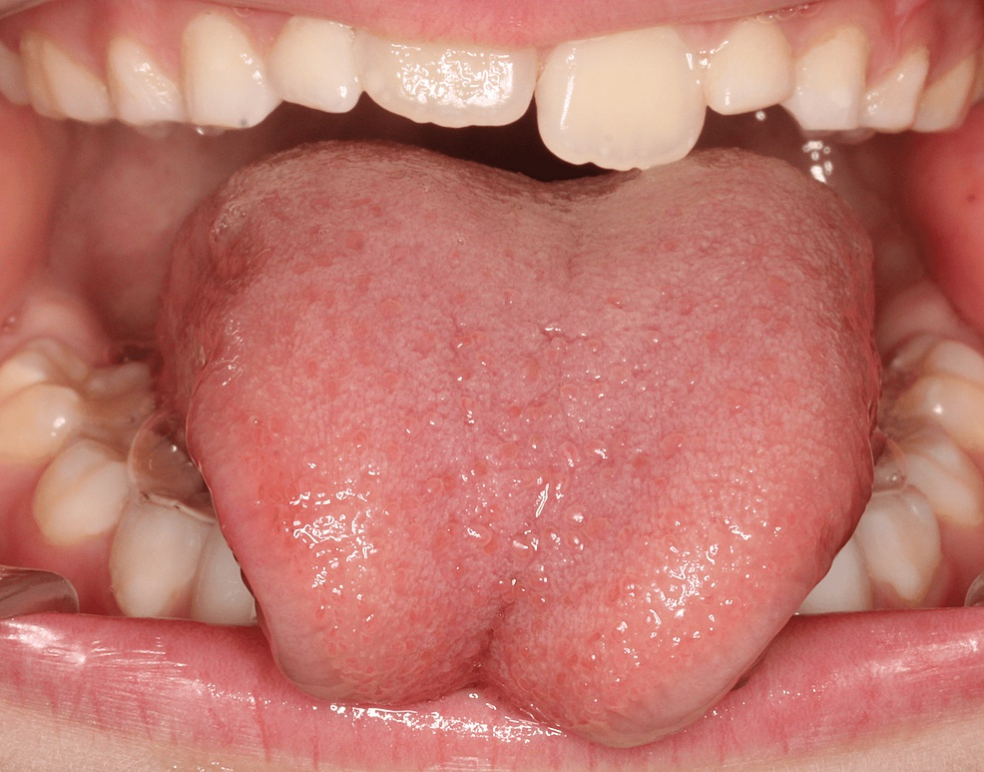
Tongue in protrusion (Case 2)

An eight-year-old Caucasian boy presented to our polyclinic in Matosinhos (Portugal) for an orthodontic evaluation. After clinical and radiographic evaluation, he was diagnosed with an anterior crossbite, as well as the presence of an altered lingual frenulum (ankyloglossia) (Figures 4, 5). The patient was referred to a speech myofunctional therapy consultant. Upon evaluation, the presence of an altered lingual frenulum was confirmed, which negatively influenced tongue mobility and functions, particularly chewing and speaking.

**Figure 6:**
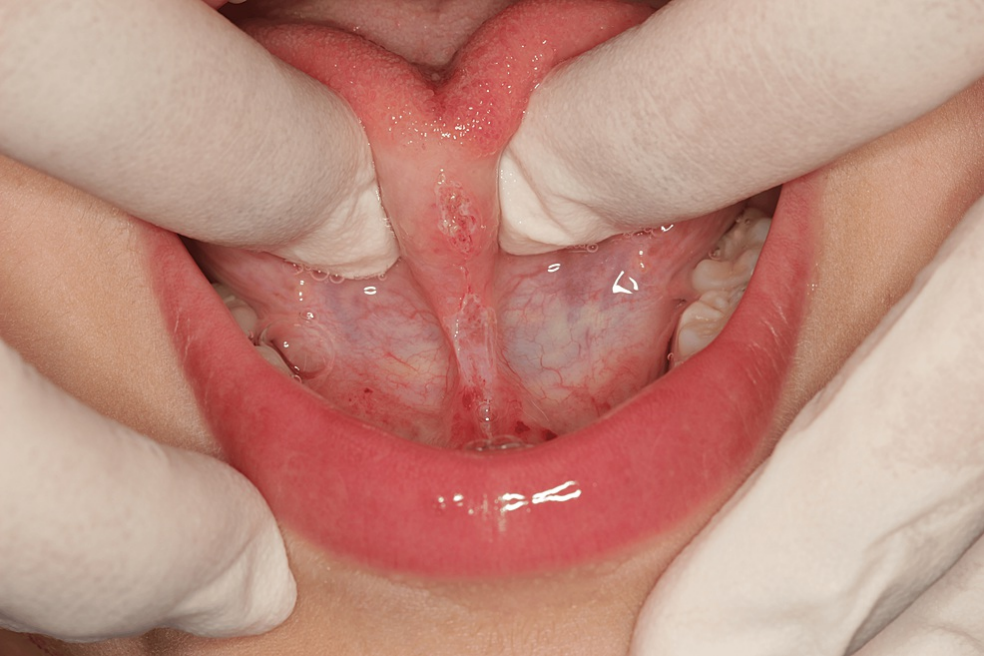
Lingual frenotomy (Case 2)

After discussing all treatment options with the family and obtaining informed consent, the decision to perform diode laser frenotomy under local anesthesia with a 2% lidocaine infiltrative solution was made. The same technique as described in case report 1 was used (Figure 6).

No sutures were needed and there were no complications during and after the surgery. Analgesic medication in the first three days (paracetamol per os), soft and cold food, and the application of a 0.12% chlorhexidine-based gel twice a day after his usual oral hygiene was recommended. The patient was referred to his speech myofunctional therapist to start post-surgical rehabilitation in the first two days and schedule a follow-up appointment after one week.

## Discussion

Ankyloglossia and breastfeeding

Dealing with breastfeeding difficulties with a neonate with ankyloglossia can be challenging for the team that supports the dyad, as even without ankyloglossia most mothers experience some difficulties in this area. A recent systematic review and meta-analysis have demonstrated that pediatric ankyloglossia is associated with suboptimal breastfeeding, infant gastroesophageal reflux, low maternal breastfeeding self-efficacy, and moderately intense nipple pain. This can deter mothers from practicing exclusive breastfeeding [3] for impeding a great breast milk transfer by the infant, thus being considered a risk factor for premature breastfeeding cessation [4,14]. In the presence of a restrictive lingual frenulum, frenotomy can increase maternal and infant comfort, improve breast milk transfer, and prevent cessation of breastfeeding. Nevertheless, the American Academy of Paediatric Dentistry emphasizes that the surgical treatment of ankyloglossia must be decided on an individual basis, by discussing the case with the family and within a multidisciplinary team of health professionals [2].

**Figure 7:**
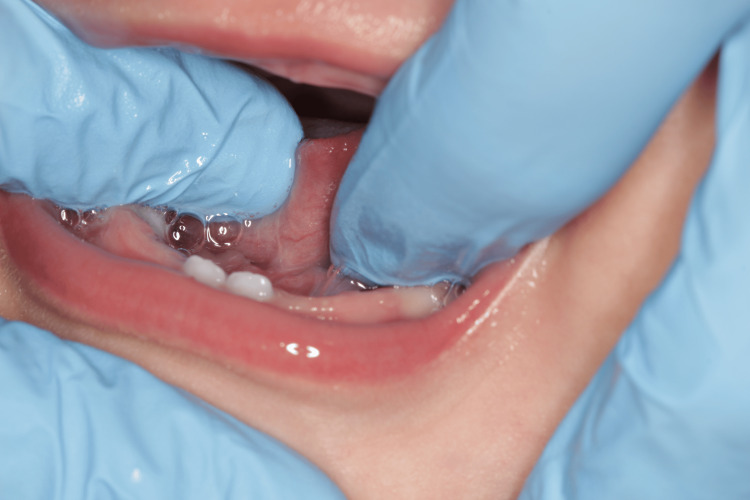
Four months after lingual frenotomy (Case 1)

Four months after surgery, when the patient was five months old, several improvements were observed regarding on breastfeeding (Figure 7). The infant continued to be exclusively breastfed, with no gastroesophageal reflux, perfect weight gain, and no mamillary or nipple pain reported by his mother. A breastfeeding session was observed by an IBCLC, and there were no more concerns about milk transfer or comfort for both baby and mother.

Ankyloglossia and malocclusion

Ankyloglossia has been related to dental malocclusion and craniofacial growth alterations. There is a clear biological plausibility for this hypothesis, and available evidence is scarce. Most ankyloglossia-related evidence is focused on breastfeeding, swallowing, and speech. However, some recent systematic reviews have highlighted the lack of quality evidence on those very aspects [15-17]. Besides genetic factors, some environmental factors have been considered responsible for influencing anterior crossbites, such as incorrect postural habits, prolonged tongue-sucking habits, low tongue posture, ankyloglossia, atypical swallowing, airway obstruction, oral breathing, hormonal imbalances, trauma, premature loss of primary teeth, congenital anatomical defects and muscular dysfunction [18-20].

**Figure 8:**
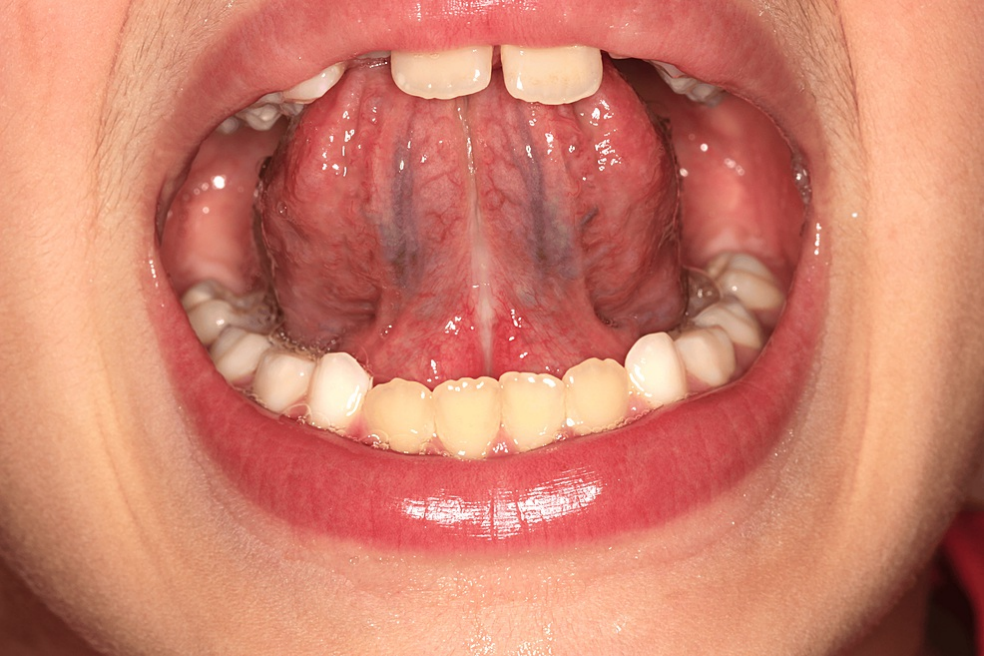
Seven months after lingual frenotomy (Case 2)

In the second case report, the eight-year-old child presented with an anterior crossbite. After evaluation by a speech myofunctional therapy specialist, a lingual frenotomy was performed. The patient is currently undergoing treatment with functional jaw orthopedics to correct the anterior crossbite and speech myofunctional therapy sessions to improve the tonus and mobility of the tongue and lips, as well as compromised oral functions. Seven months after surgery (Figure 8), the patient and his family have reported improvements in oral function, speech, chewing, swallowing, breathing, and sleep.

Surgical treatment of ankyloglossia

The treatment of ankyloglossia can be frenotomy, frenectomy, and frenuloplasty [2,13], and can be performed by a conventional method, with scissors, or electrocautery or laser techniques [2,4,14]. The use of laser technologies for frenotomies/frenectomies has been demonstrated as an effective method for the surgical treatment of ankyloglossia [2,8,14,21,22]. Different types of lasers can be used in lingual frenulum surgery. The laser wavelength selection should be based on optical affinity for hemoglobin and water (e.g., diode lasers and erbium) permit precise cutting, and provide hemostasis, and biomodulation properties, but can thermally damage surrounding tissues. Myofunctional therapy as an adjunctive to the surgical treatment of ankyloglossia can significantly improve tongue function [22].

## Conclusions

Pediatric ankyloglossia, which can lead to difficulties in breastfeeding and alterations in orofacial and oro-functional development, can be easily diagnosed at the beginning of life. Early diagnosis can prevent premature breastfeeding cessation and other problems in the future. The surgical treatment (lingual frenotomy) of this condition can be performed efficiently using laser technique. However, additional studies are necessary in this area, particularly with respect to the moment, technique, and wound healing in the lingual frenulum.
